# Supplementary material for: Work-related stress, reason for consultation and diagnosis-specific sick leave: How do they add up?
Source: PLoS One. 2023 Jul 18;18(7):e0288751. doi: 10.1371/journal.pone.0288751 (PMC10353798; doi:10.1371/journal.pone.0288751)
Supplement: Attachment — Submitted filename: Response to Reviewers.docx [file pone.0288751.s002.docx]

# Response to reviewers’ comments

PONE-D-22-26733, PLOS ONE

Work-related stress, reason for consultation and diagnosis-specific sick leave: How do they add up?

Decision received 25 November 2022

Dear Editor,

Thank you for the comments. Based on our understanding of the research field, the problem addressed in the study, and PLOS ONE style requirements, we have adjusted the manuscript accordingly. Changes have been highlighted in the manuscript. In addition, point-by-point responses to the comments are presented below.

## Comments from the academic editor

## *Comment 1: Please ensure that your manuscript meets* *PLOS ONE's style requirements, including those for file naming.*

Response: Thank you for kindly reminding us of the style requirements. The manuscript has been revised to align with the PLOS ONE style templates and the submission guidelines.

*Comment 2. We note that you have indicated that data from this study are available upon request. PLOS only allows data to be available upon request if there are legal or ethical restrictions on sharing data publicly. If there are ethical or legal restrictions on sharing a de-identified data set, please explain them in detail (e.g., data contain potentially sensitive information, data are owned by a third-party organization, etc.) and who has imposed them (e.g., an ethics committee). Please also provide contact information for a data access committee, ethics committee, or other institutional body to which data requests may be sent.*

Response: To protect privacy and confidentiality, the raw data and the datasets generated in this study are not publicly available due to restrictions stated in the ethical approval (reference number 125-5) issued by the Regional Ethical Review Board in Gothenburg, Sweden (later merged into the Swedish Ethical Review Authority, https://etikprovningsmyndigheten.se/). According to the approval, the data are to be managed carefully, and the results presented so that individuals cannot be identified. In addition, “All results from the project must be reported at group level with de-identified material” (ethical approval 125-5, section 6.3). The raw data and datasets generated cannot be made publicly available, because of the risk that individuals are identified if study data are combined. Sensitive data on work-related stress, days of sick leave and sick leave diagnosis could then be traced to the persons identified. Interested researchers may send data access requests to the corresponding author (AMH), anna-maria.hulten@gu.se, or the TIDAS research group at the Institute of neuroscience and physiology, tidas@neuro.gu.se. Please refer to the TIDAS project “Early identification of work-related stress”, study "How do they add up?", and data set AddUp2023.

## Comments from reviewer 1

*Comment 1: I would like to thank all authors for their interesting and useful work. The paper was presented in a scientific manner. Introduction, methodology, results and conclusions are clearly written. Study limitations were identified. If possible can add a flow chart. However, authors have already explained the patient recruitment procedure as well.*

Response: Thank you for the positive comments.

**Comments from reviewer 2**

*Comment 1: The text alignment should be in Justify (Ctr+J)*

Response: We have studied the style requirements and looked at several articles published in PLOS ONE, but we cannot find any requirement of adjusting the text to both left and right (justify). We therefore chose not to use this typographic alignment.

*Comment 2: Tables format/bordering should be similar and formal.*

Response: The table bordering has been adjusted.

*Comment 3: Table citation within the paragraphs should be revised.*

Response: In the paragraphs, tables are cited as “Table 1”, “Table 2” etc. We have studied the style requirements and looked at a number of articles published in PLOS ONE, but we cannot find any ground for revising the table citations.

*Comment 4: Line spacing including the headings should be revised well as PLOS requirements.*

Response: Both body text and headings have been revised.

*Comment 5: Some figure captions are bold and some are not, please update it.*

Response: The figure captions have been revised in accordance with the style requirements.

*Comment 6: Exaggerated paragraph indentation should be amended.*

Response: The indentation has been adjusted.

*Comment 7: Abbreviations should be written in full statement (General practitioners first term under the abstract written as GPs) hence, please update all this abbreviations issue.*

Response: Abbreviations have been adjusted, so that they are defined upon first appearance in the text.

*Comment 8: Was that not possible to analyse logistic regression for the associations besides the P value?*

Response: Thank you for this question. In this study, the Pearson chi-squared test and calculation of crude relative risks (RR) were considered suitable statistical methods in data analysis, based on the four research questions on the bivariate relationships between work-related stress, reason for consultation, and diagnosis-specific sick leave. Developing multivariable clinical prediction models for binary outcomes was not the scope of this study. However, multivariate analyses could have been used to analyse the three variables simultaneously. Given that non-parametric tests had to be used, binomial logistic regression could be suitable for analysing the relationship between stress, reason for consultation, and diagnosis-specific sick leave. Due to the relatively low sample size, the power was, however, considered insufficient for performing more complex analyses [1]. The analyses would include a large number of statistical tests, which would influence the sample size needed; the more tests, the larger the sample.

In addition to the above comments, minor grammatical changes and clarifications have been made to improve readability (highlighted in the file labelled 'Revised Manuscript with Track Changes').

We look forward to hearing from you regarding our submission and to respond to any further questions or comments.

Yours sincerely,

Anna-Maria Hultén, MSc, PhD student

**Reference**

1. Bujang MA, Sa'at N, Sidik T, Joo LC. Sample Size Guidelines for Logistic Regression from Observational Studies with Large Population: Emphasis on the Accuracy Between Statistics and Parameters Based on Real Life Clinical Data. Malays J Med Sci. 2018;25(4):122-30.
