# Supplementary material for: Work-related stress, reason for consultation and diagnosis-specific sick leave: How do they add up?
Source: PLoS One. 2023 Jul 18;18(7):e0288751. doi: 10.1371/journal.pone.0288751 (PMC10353798; doi:10.1371/journal.pone.0288751)
Supplement: Attachment — Submitted filename: Response to Reviewers.docx [file pone.0288751.s003.docx]

**Response to reviewers**

PONE-D-22-26733, PLOS ONE

Work-related stress, reason for consultation and diagnosis-specific sick leave: How do they add up?

Decision received 18 April 2023

Dear Editor,

Thank you for the additional comments, which we found very valuable. Changes have been highlighted in the manuscript. In addition, point-by-point responses to the comments are presented below.

**Comments from reviewer 3**

*Comment 1: I think authors did not address comment from reviewer 1. As you see, reviewer 1 asked for a flow chart. So far I understand a flow chart of the study population.*

Response: Reviewer 1’s comment on adding a flowchart read: “If possible can add a flow chart. However, authors have already explained the patient recruitment procedure as well”. Unfortunately, we gave no response to that comment. The reasoning for not including a flowchart was that the inclusion process only included a few simple steps, which we have, as stated by reviewer 1, described in words. In addition, Table 1 gives further information about the inclusion process. A flowchart of enrolment, allocation and follow-up for the RCT can be found elsewhere (1).

*Comment 2: Is the study cross-sectional or longitudinal? Although authors mention a cross-sectional and prospective longitudinal study design, I think this needs an explanation how a study can be both? Is it they wanted to see differences between two time points, but that was not the case in this study?*

Response: Thank you for the valuable comment. The study is a prospective longitudinal observational study with initial cross-sectional analyses. Changes have been made to simplify and reduce the risk of misinterpretation (p 2, row 22; p 7, row 146; p 7, row 156; p 21, row 429; p 26, row 542-543). In addition, figure 1 and 4 have been revised.

*Comment 3: How authors calculated crude relative risk - is it manually without a regression model? If so, they also calculated CI and p-value. Without having a model, the statistical results for CRR could be spurious.*

Response: Thank you for bringing this ambiguity to our attention. We did not perform regression analysis. The relative risk and confidence interval were calculated using IBM SPSS Statistics version 25.0 (Analyze >Descriptive Statistics >Crosstabs). Therefore, we chose to remove the word crude in order not to lead into the idea that the calculated risk constituted an initial step in a multivariable regression analysis (p 13, row 289; p 17, row 357; p 18, row 368).

*Comment 4: How authors made an index for WSQ instrument is not clear, and what is the reliability of the instrument in this study.*

Response: We agree with Reviewer 3 that the text on how the dichotomized WSQ values were created had to be clarified and have revised the text accordingly (p 9, row 188-206; p 10, row 207-208; p 10, row 211-212; p 10, row 217-229; p 11, row 230-235).

Thank you for also addressing the reliability of the WSQ. The questionnaire was developed based on both theory and empirical findings about work-related stressors and stress. The results of a qualitative study on women’s views of opportunities for and obstacles to returning to work (2) significantly affected the questionnaire development. As written in the manuscript, the face validity and the test-retest reliability has been tested for women and men separately (3, 4). A pilot group of ten employed working women and a pilot group of seven employed working men confirmed the face validity of the questionnaire. The confirmative responses from both women and men were based on their comments and notes on scale steps and formulation of the questions as well as if the questionnaire corresponded to their understanding of work-related stress after filling out the WSQ. The test-retest reliability of the WSQ has been assessed in two separate test–retest studies with 52 women and 41 men. The reliability was analysed with a non-parametric statistical method for evaluation of paired data (5). The analysis indicated a satisfactory reliability for both women and men.

The WSQ was used in this study, since it includes aspects relating to work, but also to the individual and the time spent out of work. In addition, it is developed to assess the risk of future long-term sick leave in a primary health care setting. At the time of the study there was no other high-quality questionnaire that included all the aspects of interest when looking at stress-related ill health from a transactional perspective. The Cronbach’s alpha is frequently used in research as a measure of scale reliability. However, there are assumption to when it can be used, for instance that the items should be continuous with normal distributions (6, 7). In our study the data is ordinal and therefore we chose not to include Cronbach’s alpha as a measure of scale reliability (internal consistency) in the manuscript.

The following articles describe the development of the WSQ as well as studies in which the questionnaire has been used (all items or in some cases selected items were used):

- Hultén et al, 2022. Work‑related stress and future sick leave in a working population seeking care at primary health care centres: a prospective longitudinal study using the WSQ (8);
- Hultqvist et al, 2021. Does a brief work-stress intervention prevent sick-leave during the following 24 months? A randomized controlled trial in Swedish primary care (9);
- Hultén et al, 2021. Self-reported sick leave following a brief preventive intervention on work-related stress: a randomised controlled trial in primary health care (1);
- Sandheimer et al, 2020. Effects of a work stress intervention on healthcare use and treatment compared to treatment as usual: A randomised controlled trial in Swedish primary healthcare (10);
- Hultén et al, 2020. Positioning work related stress - GPs' reasoning about using the WSQ combined with feedback at consultation (11);
- Bjerkeli et al, 2020. Does early identification of high work related stress affect pharmacological treatment of primary care patients? - Analysis of Swedish pharmacy dispensing data in a randomised control study (12);
- Frantz et al, 2019. The Work Stress Questionnaire (WSQ) - Reliability and face validity among male workers (4);
- Holmgren et al, 2019. Does early identification of work-related stress, combined with feedback at GP-consultation, prevent sick leave in the following 12 months? (13);
- Holmgren et al, 2016. Early identification in primary health care of people at risk for sick leave due to work-related stress - Study protocol of a randomized controlled trial (RCT) (14);
- Holmgren et al, 2014. Remain in work-What work-related factors are associated with sustainable work attendance: A general population-based study of women and men (15);
- Holmgren et al, 2013. Early identification of work-related stress predicted sickness absence in employed women with musculoskeletal or mental disorders: a prospective, longitudinal study in a primary health care setting (16);
- Holmgren et al, 2013: The combination of work organizational climate and individual work commitment predicts return to work in women but not in men (17);
- Holmgren et al, 2010. The association between poor organizational climate and high work commitments, and sickness absence in a general population of women and men (18);
- Holmgren et al, 2009. The prevalence of work-related stress, and its association with self-perceived health and sick-leave, in a population of employed Swedish women (19);
- Holmgren et al, 2009. Development of a questionnaire assessing work-related stress in women - Identifying individuals who risk being put on sick leave (3);
- Holmgren et al, 2004. Women on sickness absence - Views of possibilities and obstacles for returning to work. A focus group study (2).

*Comment 5: Author mentioned about power of the study in answering the comment of Reviewer 2. My question is to author whether they had a power calculation; moreover, I notice they have 232 subjects, in that case they are able to perform some kind of models.*

Response: Thank you for raising these questions. A power analysis was performed for the primary outcome measure of the RCT (14). No power analysis was performed for this study, since the study was based on data from the RCT and the number of participants was therefore limited to those patients participating in the trial.

In all 232 patients participated in the study. However, not all patients perceived stress. For instance, 49 patients perceived high stress due to indistinct organisation and conflicts, and 105 patients perceived stress due to individual demands and commitment (Table 5). In addition, not all patients were on sick leave with a mental or musculoskeletal diagnosis (Table 5). Therefore, for some analysis Fisher’s exact test was used instead of Pearson chi-squared test to calculate p-values (the expected cell frequency was less than 5 in more than 20% of the cells in the contingency table). Due to these circumstances, the power would have been to low to perform multivariable analysis.

*Comment 6: Authors mention about RCT in method - I understand they were explaining how the data were collected, but it seems they talk so much about RCT. Readers may get confused.*

Response: Thank you for the valuable comment. We have now described the RCT in less detail and rearranged the text as well (p 7, row 149-165; p 8, 166-170).

In addition to the above comments, minor grammatical changes and clarifications have been made to improve readability (highlighted in the file labelled 'Revised Manuscript with Track Changes').

We look forward to hearing from you regarding our submission and to respond to any further questions or comments.

Yours sincerely,

Anna-Maria Hultén, MSc, PhD student

**References**

1.	Hultén AM, Bjerkeli P, Holmgren K. Self-reported sick leave following a brief preventive intervention on work-related stress: A randomised controlled trial in primary health care. BMJ Open. 2021;11(3).

2.	Holmgren K, Dahlin Ivanoff S. Women on sickness absence--views of possibilities and obstacles for returning to work. A focus group study. Disabil Rehabil. 2004;26(4):213-22.

3.	Holmgren K, Hensing G, Dahlin-Ivanoff S. Development of a questionnaire assessing work-related stress in women - Identifying individuals who risk being put on sick leave. Disabil Rehabil. 2009;31(4):284-92.

4.	Frantz A, Holmgren K. The Work Stress Questionnaire (WSQ) - Reliability and face validity among male workers. BMC Public Health. 2019;19(1).

5.	Svensson E. Ordinal invariant measures for individual and group changes in ordered categorical data. Stat Med. 1998;17(24):2923-36.

6.	McNeish D. Thanks coefficient alpha, we'll take it from here. Psychol Methods. 2018;23(3):412-33.

7.	Gadermann AM, Guhn M, Zumbo BD. Estimating Ordinal Reliability for Likert-Type and Ordinal Item Response Data: A Conceptual, Empirical, and Practical Guide. Pract Assess Res Eval. 2012;17:1-13.

8.	Hultén AM, Bjerkeli P, Holmgren K. Work-related stress and future sick leave in a working population seeking care at primary health care centres: a prospective longitudinal study using the WSQ. BMC Public Health. 2022;22(1).

9.	Hultqvist J, Bjerkeli P, Hensing G, Holmgren K. Does a brief work-stress intervention prevent sick-leave during the following 24 months? A randomized controlled trial in Swedish primary care. Work. 2021;70(4):1141-50.

10.	Sandheimer C, Hedenrud T, Hensing G, Holmgren K. Effects of a work stress intervention on healthcare use and treatment compared to treatment as usual: a randomised controlled trial in Swedish primary healthcare. BMC Fam Pract. 2020;21(1):133.

11.	Hultén AM, Dahlin-Ivanoff S, Holmgren K. Positioning work related stress - GPs' reasoning about using the WSQ combined with feedback at consultation. BMC Fam Pract. 2020;21(1):187.

12.	Bjerkeli PJ, Skoglund I, Holmgren K. Does early identification of high work related stress affect pharmacological treatment of primary care patients? - analysis of Swedish pharmacy dispensing data in a randomised control study. BMC Fam Pract. 2020;21(1):70.

13.	Holmgren K, Hensing G, Bültmann U, Hadzibajramovic E, Larsson MEH. Does early identification of work-related stress, combined with feedback at GP-consultation, prevent sick leave in the following 12 months?: a randomized controlled trial in primary health care. BMC Public Health. 2019;19(1):1471-2458.

14.	Holmgren K, Sandheimer C, Mardby AC, Larsson ME, Bultmann U, Hange D, et al. Early identification in primary health care of people at risk for sick leave due to work-related stress - study protocol of a randomized controlled trial (RCT). BMC Public Health. 2016;16(1):1193.

15.	Holmgren K, Löve J, Mårdby AC, Hensing G. Remain in work--what work-related factors are associated with sustainable work attendance: a general population-based study of women and men. J Occup Environ Med. 2014;56(3):235-42.

16.	Holmgren K, Fjällström-Lundgren M, Hensing G. Early identification of work-related stress predicted sickness absence in employed women with musculoskeletal or mental disorders: a prospective, longitudinal study in a primary health care setting. Disabil Rehabil. 2013;35(5):418-26.

17.	Holmgren K, Ekbladh E, Hensing G, Dellve L. The combination of work organizational climate and individual work commitment predicts return to work in women but not in men. J Occup Environ Med. 2013;55(2):121-7.

18.	Holmgren K, Hensing G, Dellve L. The association between poor organizational climate and high work commitments, and sickness absence in a general population of women and men. J Occup Environ Med. 2010;52(12):1179-85.

19.	Holmgren K, Dahlin-Ivanoff S, Björkelund C, Hensing G. The prevalence of work-related stress, and its association with self-perceived health and sick-leave, in a population of employed Swedish women. BMC Public Health. 2009;9;73.
